# Emergency Department Procedural Sedation Versus Operating-Theater General Anesthesia for Closed Reduction of Pediatric Forearm Fractures: A Systematic Review

**DOI:** 10.7759/cureus.115112

**Published:** 2026-08-24

**Authors:** Ahmed Al-Sum, Wesam H Aridhi, Abdulrahim J Sahli, Yasir M Darbeshi, Roaa Z Alghamdi, Muath A Alshahrani, Fesal S Alatawi, Abdulhadi A Alobud, Ayman A Khiswi, Raed S Alruwaili, Meshal F Alqubali

**Affiliations:** 1 Department of Emergency Medicine and Critical Care, King Fahad Central Hospital, Jazan, SAU; 2 College of Medicine, Jazan University, Jazan, SAU; 3 Department of General Practice, Ibn Sina National College, Jeddah, SAU; 4 College of Medicine, Bisha University, Bisha, SAU; 5 Faculty of Medicine, Tabuk University, Tabuk, SAU; 6 Faculty of Medicine, Qassim University, Qassim, SAU; 7 Faculty of Medicine, Jazan University, Jazan, SAU; 8 College of Medicine, King Faisal University, Al Ahsa, SAU

**Keywords:** clinical outcomes, closed reduction, emergency department procedural sedation, general anesthesia, operating theater, pediatric forearm fractures, resource utilization, systematic review

## Abstract

Displaced pediatric forearm fractures requiring closed reduction are traditionally managed in the operating theater under general anesthesia (GA). However, emergency department (ED)-based procedural sedation may reduce treatment delays, admissions, and resource utilization. This systematic review evaluated clinical outcomes, safety, repeat intervention, and resource implications of ED procedural sedation compared with theater-based GA and identified factors associated with unsuccessful ED management. A systematic review was conducted in accordance with the Preferred Reporting Items for Systematic Reviews and Meta-Analyses (PRISMA) 2020 statement. MEDLINE, the Cochrane Library, Web of Science, and Scopus were searched from 2000 to June 2026. Studies evaluating children with displaced or angulated forearm fractures undergoing closed reduction with ED sedation or theater-based GA were eligible. Risk of bias was assessed using the Risk of Bias in Non-randomized Studies of Interventions (ROBINS-I) and Quality in Prognosis Studies (QUIPS) tools. Due to substantial clinical and methodological heterogeneity, findings were synthesized narratively. Six observational studies met the inclusion criteria, including four direct comparative studies and two analytical observational studies. No randomized controlled trials were identified. The four comparative studies included a total of 958 children and evaluated various ED sedation approaches, including ketamine, nitrous oxide-based regimens, and opioid combinations. Most studies were at high risk of bias, primarily due to confounding by fracture severity, treatment selection, and differences in resources between ED and theater settings. ED and theater management demonstrated similar initial reductions in quality and cast-quality outcomes across the available studies. Evidence regarding redisplacement and repeat interventions was inconsistent: one study reported higher remanipulation rates after ED reduction, whereas others found no significant differences. ED management was consistently associated with shorter treatment times, reduced admission, shorter length of stay, and lower resource utilization. Factors associated with unsuccessful ED reduction included delayed presentation, non-distal fracture location, and increasing age. Implementation of a structured ED pathway was associated with higher ED treatment rates and fewer failed reductions, although causal inference was limited by the study design. ED procedural sedation appears to be a feasible option for selected pediatric forearm fractures requiring closed reduction, with potential benefits in efficiency and resource use. However, current evidence remains limited by observational study designs, selection bias, and heterogeneous treatment protocols. Further prospective studies are required to establish optimal patient selection criteria, safety, and the comparative effectiveness of ED- and theater-based management.

## Introduction and background

Forearm fractures are among the most common musculoskeletal injuries in children and represent a substantial proportion of pediatric emergency and orthopedic practice. Distal forearm fractures account for a large proportion of these injuries, with the highest incidence occurring in school-aged children and early adolescents, particularly boys [1-4]. While many fractures can be managed with immobilization alone, displaced, angulated, or translated fractures may require reduction to restore acceptable alignment [2,5].

Children have greater remodeling potential than adults, allowing some residual deformity after fracture. However, acceptable alignment depends on age, remaining growth, fracture location, fracture pattern, displacement, and deformity plane [2-6]. Distal fractures generally remodel better than diaphyseal or proximal injuries, whereas older children and fractures further from the growth plate have less tolerance for residual deformity [1,5]. For displaced but reducible fractures, closed reduction and cast immobilization remain standard treatment. The quality of reduction and cast stability are important factors because loss of alignment may require repeat manipulation, operative fixation, additional imaging, and further anesthetic exposure [3,5,6].

Traditionally, displaced pediatric forearm fractures have been reduced in the operating theater under general anesthesia (GA). This approach provides a controlled environment with muscle relaxation, fluoroscopic assessment, specialist support, and immediate access to fixation if required [3-7]. However, theater-based management may involve delays, fasting, admission, increased resource use, and greater logistical burden for children, families, and healthcare systems. GA also carries perioperative risks.

Advances in pediatric procedural sedation have enabled many fracture reductions to be performed in the emergency department (ED). Common approaches include nitrous oxide, ketamine, opioid-based regimens, hematoma block, and combined techniques [3-6]. When delivered by trained staff with appropriate monitoring and resuscitation facilities, ED procedural sedation can allow timely reduction and the potential for same-day discharge [1-7]. However, sedation-related adverse events remain possible, and safe practice depends on patient selection, staff expertise, monitoring standards, and airway-management capability [1,3,6].

Clinical guidance increasingly supports ED-based reduction of suitable pediatric forearm fractures where appropriate. Uncertainty remains about whether ED reduction yields outcomes comparable to those of theater-based manipulation [3-8]. Potential benefits of ED management include reduced waiting times, hospital admissions, lengths of stay, and theater utilization. These advantages must be balanced against concerns regarding reduced quality, loss of alignment, repeated manipulation, and the subsequent need for GA [4-7]. Assessment of reduction may also be limited in ED settings where fluoroscopy is unavailable. Furthermore, direct comparisons between ED and theater pathways are complicated because more complex, unstable, or severely displaced fractures are often preferentially managed in theater, potentially creating confounding by indication [6-12].

Existing comparative studies have reported inconsistent findings regarding repeat intervention, redisplacement, and treatment failure after ED sedation versus theater-based GA [3-7]. Differences in sedation methods, fracture selection, reduction techniques, imaging availability, casting methods, and follow-up protocols contribute to variability between studies. Observational evidence suggests that factors such as older age, delayed presentation, non-distal fracture location, and complete translation may increase the likelihood of unsuccessful ED management.

This systematic review aimed to evaluate the clinical outcomes, safety, repeat-intervention requirements, and resource implications of ED procedural sedation compared with theater-based GA for closed reduction of pediatric forearm fractures. A secondary aim was to identify factors associated with unsuccessful ED reduction and the subsequent need for theater management.

## Review

Methods

Review Design and Questions

A systematic review of comparative and analytical observational studies was conducted to evaluate ED procedural sedation and operating-theater GA for the closed reduction of pediatric forearm fractures. The review was reported in accordance with the Preferred Reporting Items for Systematic Reviews and Meta-Analyses 2020 (PRISMA 2020) statement [13].

The primary review question was whether ED procedural sedation and theater-based GA differ in clinical outcomes, safety, and resource use for children requiring closed reduction of forearm fractures. The secondary question examined patient, fracture, presentation, and service-level factors associated with unsuccessful ED reduction or subsequent theater intervention.

Eligibility Criteria

Studies were eligible if they included children younger than 18 years with displaced, angulated, or translated fractures of the radius, ulna, or both bones of the forearm that required closed reduction. Eligible fracture locations included distal, metaphyseal, physeal, diaphyseal, and two-bone fractures. Studies with mixed pediatric fracture populations were included only when forearm-specific data could be extracted.

Eligible interventions included ED-based closed reduction using procedural sedation, conscious sedation, procedural analgesia, or other structured non-theater pathways, including nitrous oxide, ketamine, opioid-based regimens, hematoma block, or combined approaches. For comparative studies, the comparator was closed reduction under GA in an operating theater or equivalent setting. Analytical studies evaluating predictors of ED treatment failure were also eligible.

Outcomes included reduction success, loss of reduction, redisplacement, repeat manipulation, subsequent intervention, unplanned GA or theater transfer, surgical fixation, cast-quality measures, residual deformity, fracture union, procedural or anesthetic complications, neurovascular complications, treatment timing, admission, length of stay, discharge status, theater utilization, cost, patient or caregiver experience, and predictors of unsuccessful ED reduction. Outcome definitions were accepted as reported by individual studies.

Eligible designs included randomized and non-randomized controlled trials, comparative cohort studies, controlled before-and-after studies, case-control studies, and analytical observational studies. Single-cohort studies were included only when they provided analytical evaluation of relevant predictors or outcomes. Exclusion criteria were case reports, non-analytical case series or audits, reviews, guidelines, insufficient conference abstracts, studies without reduction outcomes, adult or non-separable mixed-age populations, non-forearm injuries, open or pathological fractures, major multitrauma, and fractures requiring immediate operative fixation.

Search Strategy and Information Sources

Four electronic databases were searched in June 2026: MEDLINE via PubMed, the Cochrane Library, Web of Science, and Scopus. Searches included records published between 2000 and 2026. Reference lists of included studies and relevant reviews were also screened.

Search terms combined concepts relating to children, forearm fractures, closed reduction, ED sedation or analgesia, and theater-based GA. The search strategy was adapted for each database using appropriate field codes and indexing terms.

Study Selection and Data Extraction

Records were imported into Zotero (Corporation for Digital Scholarship, Vienna, VA, USA), and duplicates were removed through automated and manual checks. Titles and abstracts were screened independently by two reviewers, followed by independent full-text assessment of potentially eligible studies. Disagreements were resolved through discussion and, when required, consultation with a third reviewer. Study selection was presented using a PRISMA 2020 flow diagram. Data were independently extracted by two reviewers using a standardized, piloted form. Extracted data included study characteristics, participant demographics, fracture characteristics, intervention and comparator details, sedation and analgesia protocols, monitoring, fluoroscopy use, casting methods, follow-up, outcome definitions, treatment outcomes, complications, resource use, and information required for risk-of-bias assessment. Numerical findings were extracted as reported. Reviewer-calculated percentages were labeled accordingly. Reported statistical measures, including confidence intervals (CIs) and p-values, were retained in their original formats. Ambiguities or inconsistencies in definitions, denominators, or reported outcomes were recorded rather than retrospectively resolved. Studies were grouped into two prespecified evidence streams: direct comparative studies evaluating ED reduction with procedural sedation versus theater reduction with GA and analytical observational studies evaluating predictors of ED treatment failure or the impact of ED treatment pathways. These evidence streams were synthesized separately.

Risk-of-Bias Assessment

Risk of bias in intervention and exposure studies was assessed independently by two reviewers using the Risk of Bias In Non-randomized Studies of Interventions (ROBINS-I) tool [14]. Particular attention was given to confounding by fracture severity, treatment selection, differences in sedation and imaging resources, operator experience, incomplete follow-up, and outcome-assessment bias. Judgments were categorized as low, moderate, serious, critical, or no information.

Studies evaluating prognostic factors for unsuccessful ED reduction were assessed using the Quality in Prognosis Studies (QUIPS) tool [15]. Domains were classified as low, moderate, or high risk of bias. Disagreements were resolved through discussion or adjudication by a third reviewer. No numerical quality scores were calculated.

Data Synthesis

Meta-analysis was not performed because studies differed substantially in design, treatment allocation, sedation protocols, fracture characteristics, imaging availability, casting methods, outcome definitions, and follow-up. Studies were grouped by evidence stream and outcome domain. Comparative outcomes included reduction success, loss of reduction, repeat intervention, cast quality, complications, admission, length of stay, treatment timing, and resource use. Analytical outcomes included predictors of unsuccessful ED reduction, subsequent theater intervention, pathway implementation effects, and resource implications. For each outcome, findings were summarized using reported definitions, sample sizes, effect estimates, CIs, and statistical significance. Differences between studies were interpreted in relation to clinical and methodological heterogeneity, including fracture complexity, sedation approach, fluoroscopy availability, clinician expertise, and risk of bias. Non-significant results were interpreted as an absence of evidence for a difference rather than evidence of equivalence.

Results

Study Selection

**Figure 1:**
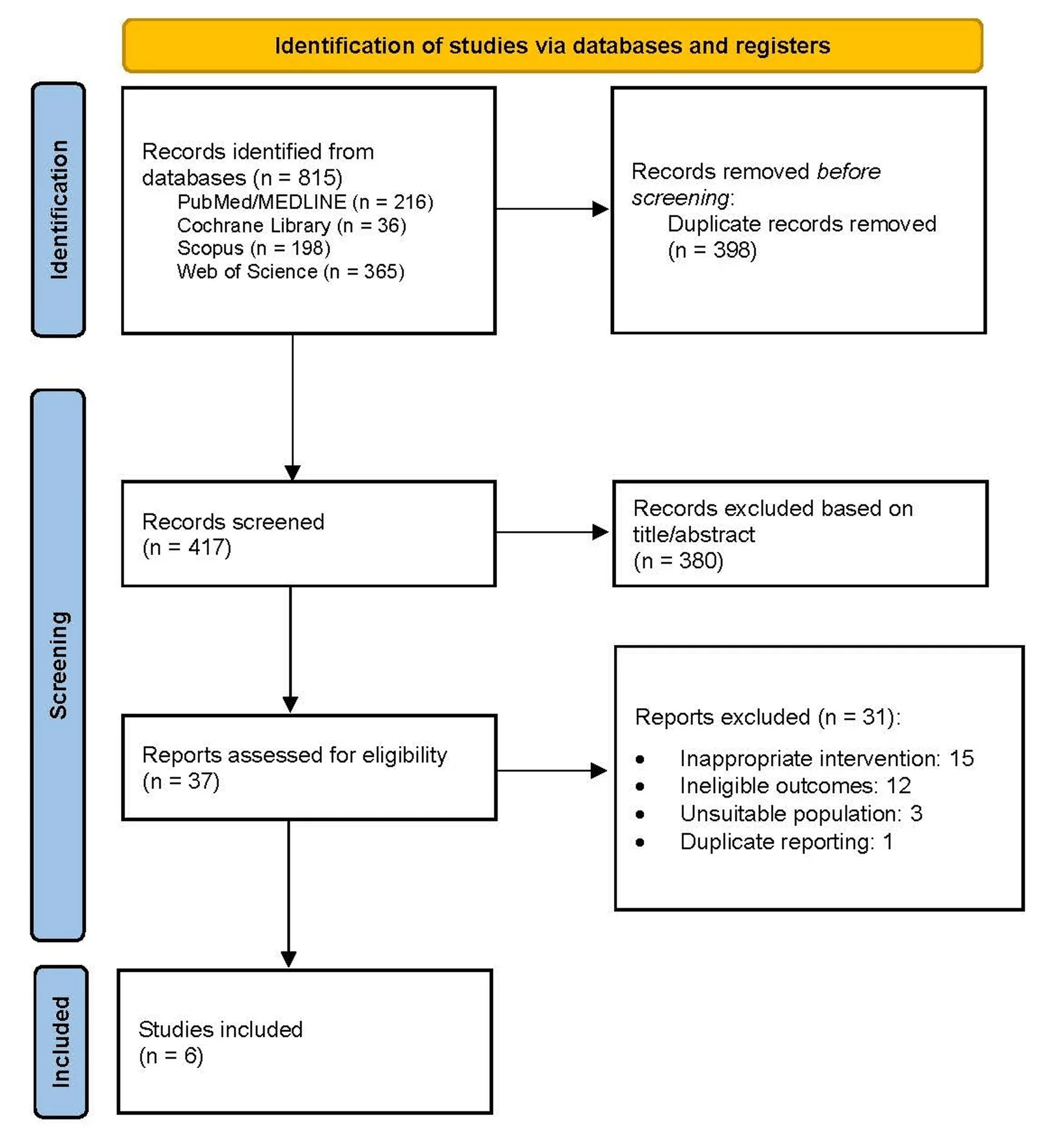
PRISMA 2020 flow diagram depicting the study selection process for the systematic review Following the identification of records through database searching and other sources, duplicates were removed, and the remaining records were screened. Full-text articles were assessed for eligibility, and exclusions were documented with reasons. Studies meeting the inclusion criteria were included in the final synthesis. PRISMA: Preferred Reporting Items for Systematic Reviews and Meta-Analyses

The database search identified 815 records. After removal of 398 duplicates, 417 records underwent title and abstract screening. Thirty-seven reports were assessed in full text; six studies met the eligibility criteria and were included in the review. Four studies directly compared ED procedural sedation with operating-theater GA for closed reduction of pediatric forearm fractures, while two studies evaluated predictors of ED treatment failure or the impact of an ED management pathway [1-6]. No randomized controlled trials were identified; all included studies were observational (Figure 1).

The six studies were published between 2011 and 2023 and included populations from New Zealand, the United Kingdom, Ireland, and Australia. Across the comparative studies, ED reduction was performed using various sedation protocols, including ketamine, nitrous oxide-based regimens, and opioid combinations, whereas theater-based reduction was performed under GA [1-4]. The two analytical observational studies assessed risk factors for subsequent theater intervention after ED reduction and outcomes following implementation of a structured ED pathway [5,6].

Study Characteristics

The four comparative studies included a total of 958 children with displaced or angulated forearm fractures. Eligibility for ED management generally excluded more complex injuries, including completely displaced fractures, fractures requiring fixation, or cases with unacceptable alignment after initial assessment. Theater cohorts frequently included more complex or severe fracture patterns. Fluoroscopy or image intensifier guidance was available for theater procedures but was not routinely available for ED reductions (Table 1) [1-4].

**Table 1:** Characteristics of included studies Characteristics of the six included studies evaluating ED-based management of pediatric forearm fractures requiring closed reduction. Data are presented according to study design, evidence stream, setting, participant characteristics, intervention details, comparator groups, and follow-up methods. Stream 1 refers to direct comparative studies of ED sedation versus theater-based GA. Stream 2 refers to analytical observational studies addressing predictors of ED treatment failure or pathway implementation and was synthesized separately from direct comparisons. None of the included studies were randomized controlled trials ED: emergency department, GA: general anesthesia, IQR: interquartile range, QUIPS: Quality in Prognosis Studies; ROBINS-I: Risk of Bias in Non-randomized Studies of Interventions, SD: standard deviation

Characteristic	Country/setting	Design/evidence stream/risk-of-bias assessment tool	Recruitment period	Sample/groups (n)	Age (mean/median)	Sex (male/female)	Fracture types/key locations	Principal inclusion criteria	Principal exclusion criteria	Sedative/analgesic agent (ED)	Personnel (sedation/reduction)	Fluoroscopy/image intensifier	Casting method	Follow-up schedule/duration
Betham et al., 2011 [1]	New Zealand; Waikato Hospital, Hamilton (university-affiliated teaching hospital)	Comparative cohort (prospective ED, retrospective theater); Stream 1; ROBINS-I	January-December 2009	174 of 385 presentations; ED 108 vs GA 66	ED: 7.0 years (SD 2.7) vs GA: 8.6 years (SD 2.9)	ED: 67:41; GA: 35:31	Radius and/or ulna ± dislocation; Salter-Harris type I/II, distal and mid/proximal shaft, radial head/neck, Monteggia	Pediatric forearm fracture requiring manipulation before casting	Open fractures; complete displacement; need for fixation; distal humerus; carpus	Not reported (procedural sedation)	Sedationist (mostly ED); reduction by ED doctors (83%) or orthopedic doctors (17%)	Not available in ED; theater use not reported	Plaster-of-Paris cast, molded	Six-month chart review
McKenna et al., 2012 [2]	Ireland; Children’s University Hospital, Dublin (tertiary pediatric referral hospital)	Retrospective comparative cohort; Stream 1; ROBINS-I	April-October 2009	55; ED 28 vs GA 27	ED: 8.2 years (95% CI 7.1-9.3) vs GA: 7.0 years (95% CI 6.1-7.8)	ED: 20:8; GA: 16:11	Non-physeal diaphyseal and distal metaphyseal radius/ulna; greenstick and complete fractures	Displaced/angulated non-physeal forearm fracture requiring manipulation	Open fractures; physeal fractures; neurovascular compromise; initial metalwork	50% nitrous oxide (Entonox) + intravenous morphine + cyclizine	Orthopedic resident supervised; ED nurse administered Entonox; reduction by orthopedic resident	Not used in ED; used in theater	Long-arm molded plaster cast	Review at 7-10 days and 4 weeks; casts maintained for 6 weeks
Jordan et al., 2016 [3]	United Kingdom; University Hospitals Coventry and Warwickshire (teaching hospital)	Retrospective comparative cohort; Stream 1; ROBINS-I	October 2014-October 2015	66; ED (ketamine) 31 vs GA 35	Ketamine: 9.0 years (7.7-10.2) vs GA: 8.6 years (7.7-9.5)	Ketamine: 23:8; GA: 18:17	Angulated/displaced distal radius or forearm fractures (wrist and forearm)	Angulated/displaced distal radius or forearm fracture requiring manipulation	Open fractures; complete displacement; initial fixation (K-wire, nail, or plate)	Intravenous ketamine	Doctor trained in ketamine and pediatric airway management; reduction by orthopedic trainee (≥4 years), usually independently	Not used in ED; used in theater	Full molded plaster-of-Paris cast	Radiograph at 5-10 days; further review at 3-6 weeks
Chan et al., 2020 [4]	Australia; the Children’s Hospital at Westmead, Sydney (tertiary pediatric hospital)	Retrospective comparative cohort with historical protocol change; Stream 1; ROBINS-I	GA: March 2009-September 2010; nitrous oxide: September 2014–December 2015	663; ED (nitrous oxide) 301 vs GA 362	Nitrous oxide: 8.4 years (range 1-15) vs GA: 8.6 years (range 1-16)	Nitrous oxide: 208:93; GA: 230:132	Angulated metaphyseal, off-ended metaphyseal, physeal, and diaphyseal fractures	Forearm fracture undergoing closed reduction under nitrous oxide/fentanyl or GA	Pathological/multitrauma fractures; open fractures; off-ended diaphyseal fractures; Galeazzi/Monteggia fractures; missing radiographs	Nitrous oxide + intranasal fentanyl	Accredited nurse; reduction by orthopedic trainee/fellow	Not used in ED; used in theater	Circumferential molded plaster or above-elbow slabs	Radiograph at 1 and/or 2 weeks; review at 6 weeks
Seefried et al., 2022 [5]	New Zealand; National Children’s Hospital, Auckland (specialist children’s ED)	Retrospective analytical prognostic-factor cohort; Stream 2; QUIPS	1 January-30 June 2019	267 of 309 presentations; successful ED management 225 vs subsequent GA 42	Successful ED: 8.0 years (IQR 6.1-10.9) vs GA: 10.2 years (IQR 6.5-12.6)	Successful ED: 126:99; GA: 25:17	Radius and/or ulna fractures (distal, midshaft, proximal); isolated upper limb fractures	Isolated upper-limb radius/ulna fracture reduced in ED under conscious sedation	Planned theater management; unsafe sedation; supracondylar, olecranon, or radial-neck fractures; Monteggia fractures	Conscious sedation; agent not reported	Not reported	Not used (ED; no fluoroscopy)	Cast immobilization (technique not reported)	Not specified (chart review)
Fink et al., 2023 [6]	United Kingdom; Gloucestershire Royal Hospital (district general hospital)	Retrospective controlled before-and-after cohort with audit; Stream 2; ROBINS-I	Pre-COVID-19: August 2019-January 2020; post-pathway: August 2021-January 2022	159; pre-COVID-19 72 vs post-pathway 87	9.2 years (2019-2020) vs 9.8 years (2021-2022)	41:31 vs 51:36	Salter-Harris, metaphyseal (including off-ended), and greenstick fractures	Angulated/displaced wrist or forearm fracture requiring closed reduction	Open fractures; neurovascular injury; torus/undisplaced fractures; Monteggia/Galeazzi fractures; complete shaft fractures; ipsilateral humerus fracture; >1 week old; CRAFFT participants	Diamorphine + Entonox (off-ended cases); full regimen not reported	Not reported (defined within pathway)	Not reported	Casting according to pathway	Clinic review (mean 8.25 days post-pathway); minimum 11-month record review

Outcome definitions and follow-up protocols varied substantially between studies. Only one comparative study used blinded radiographic assessment of reduction outcomes, while the remaining studies relied on clinical or radiographic assessment by treating teams [2]. Follow-up periods ranged from short-term assessment of redisplacement to longer retrospective chart review (Table 2) [1-4].

**Table 2:** Outcomes and results of included studies Summary of outcome measures and reported results from the six included studies. Outcomes are presented using study-specific definitions and are not pooled due to differences in study designs, intervention protocols, outcome definitions, and follow-up periods. Values are reported as numerator/denominator (percentage), mean ± SD, or median (interquartile range) as provided in the original studies. Stream 1 studies represent direct comparisons between ED sedation and theater GA. Stream 2 studies represent single-cohort or before-and-after analyses and should not be interpreted as direct ED-versus-GA comparisons. All reported p-values are unadjusted unless otherwise specified. ED: emergency department, GA: general anesthesia, IQR: interquartile range, NS: not significant, NZ$: New Zealand dollars, OR: odds ratio, SD: standard deviation, BOAST: British Orthopaedic Association Standards for Trauma and Orthopaedics

Study ID/outcome		ED/intervention value	GA/comparator value	p-value
Betham et al., 2011 [1]	Baseline differences	Younger age; more Salter-Harris II fractures	-	0.0003/0.017
	Failed ED manipulation	4/108 (4%)	Not applicable	-
	Repeat intervention	16/108 (15%)	14/66 (21%)	0.305
	Compartment syndrome	0/108	0/66	-
	Time to manipulation	58 ± 38 min	558 ± 368 min	<0.0001
	Length of stay	139 ± 70 min	1452 ± 544 min	<0.0001
	Admission	13/108 (12%)	66/66 (100%)	<0.0001
	Modelled cost	>NZ$50,000 for overnight admission	-	-
McKenna et al., 2012 [2]	Initial angulation	22.2°	34.1°	0.003
	Initial reduction quality	~80% anatomic; ~20% acceptable	~80% anatomic; ~20% acceptable	NS
	Redisplacement requiring remanipulation	9/28 (32.1%)	3/27 (11.1%)	0.048
	Cast indices	Cast 0.79; padding 0.20; canterbury 0.99	Cast 0.80; padding 0.18; canterbury 0.98	NS
Jordan et al., 2016 [3]	Acceptable reduction	20/31 (61%)	26/35 (74%)	0.39
	Redisplacement	4/31 (12.9%)	5/35 (14.3%)	0.87
	Further surgery	2 remanipulations	1 K-wire fixation	0.48
	Cast/Gap Index	0.81/0.25	0.77/0.23	0.14/0.21
Chan et al., 2020 [4]	Baseline fracture pattern	Fewer off-ended metaphyseal fractures	More off-ended metaphyseal fractures	0.001
	Secondary procedure	23/301 (7.6%)	18/362 (5.0%)	0.155
	Loss of reduction	27/301 (9.0%)	41/362 (11.3%)	0.32
	Failed initial reduction	11 (3.7%)	0	-
	Complications	0.169	0.133	0.185
Seefried et al., 2022 [5]	Subsequent theater GA	42/267 (15.7%)	-	-
	Predictors of theater GA	Delayed presentation OR 12.9; non-distal fracture OR 7.5; age OR 1.13/year	-	0.001/0.001/0.04
	Length of stay	5.1 h (IQR 4.2-6.1)	8.5 h (IQR 5.8-23.6)	<0.001
	Modelled cost	NZ$2,885 per GA case	NZ$60,587 saved if GA halved	-
Fink et al., 2023 [6]	ED manipulation attempt	23/72 (31.9%)	73/87 (83.9%) after pathway	<0.00001
	Failed ED reduction	11/23 (47.8%)	10/73 (13.7%) after pathway	0.0013
	theater resources saved	-	~63 h and ~£75,600 over 6 months	-
	BOAST compliance	-	Neurovascular 93.1%; consent 73.6%; radiographs 100%; review ≤7 days 49.4%	-

The prognostic study included 267 children undergoing ED reduction and evaluated factors associated with subsequent theater GA. The pathway study compared ED fracture management before and after the implementation of a British Orthopaedic Association Standards for Trauma (BOAST)-aligned pathway, with staff education [5,6].

Risk of Bias Assessment

All five studies assessed using the ROBINS-I tool were judged to have a serious overall risk of bias [1-4,6]. The main limitations were confounding and selection bias, as treatment allocation was influenced by fracture complexity, clinician preference, resource availability, and local practice patterns. The most complex fractures were generally excluded from ED pathways, resulting in baseline differences between treatment groups. The prognostic study, assessed using the QUIPS tool, was judged to have a moderate risk of bias, mainly due to retrospective data collection, unblinded outcome assessment, and residual confounding from factors such as casting quality (Table 3) [5].

**Table 3:** Risk-of-bias assessment of included studies Risk-of-bias assessment of the six included observational studies using the appropriate validated appraisal tool. Five studies evaluating interventions or exposures were assessed using the ROBINS-I tool, and one prognostic-factor study was assessed using the QUIPS tool. Overall judgments were classified as low, moderate, serious, critical, or no information according to the respective tool guidance. No numerical risk-of-bias scores were calculated, and domain judgments were not summed to generate overall scores. ED: emergency department, GA: general anesthesia, QUIPS: Quality in Prognosis Studies, ROBINS-I: Risk of Bias in Non-randomized Studies of Interventions

Study ID	Tool	Overall judgement	Key concerns
Betham et al., 2011 [1]	ROBINS-I	Serious	Confounding due to non-random treatment allocation, with more complex fractures preferentially managed in theater; differential case ascertainment (prospective ED vs retrospective theater cohort); lack of adjustment for baseline differences.
McKenna et al., 2012 [2]	ROBINS-I	Serious	Confounding from clinician/parent treatment selection and greater baseline angulation in the GA group; exclusion of unstable fractures from ED management; lack of adjusted analysis. Blinded radiographic outcome assessment was a methodological strength.
Jordan et al., 2016 [3]	ROBINS-I	Serious	Treatment allocation influenced by clinician judgment and resource availability; theater group had additional resources (consultant supervision and image intensifier); retrospective design and unblinded outcome assessment.
Chan et al., 2020 [4]	ROBINS-I	Serious	Historical comparison between different treatment eras; baseline fracture-pattern imbalance favoring the ED group; selection bias and lack of adjustment; differences in imaging and casting practices between groups.
Seefried et al., 2022 [5]	QUIPS	Moderate	Retrospective prognostic cohort with limitations in participant selection, outcome assessment, and residual confounding. Casting quality could not be incorporated into the predictive model; no external validation was performed.
Fink et al., 2023 [6]	ROBINS-I	Serious	Before-and-after design affected by COVID-19-related practice changes, staff education, and increasing experience; pathway effect cannot be separated from co-interventions; unblinded assessment and modeled economic outcomes.

Initial Reduction Quality

Two comparative studies reported initial reduction quality. Neither found a significant difference between ED sedation and theater GA. One study reported similar proportions of anatomic or acceptable reductions between groups, while another found acceptable initial reduction in 61% of ED reductions compared with 74% of theater reductions (p = 0.39) [2,3]. These findings were limited by small sample sizes and serious risk of bias.

Loss of Reduction and Redisplacement

Three comparative studies reported redisplacement outcomes. Findings were inconsistent. One study reported a higher rate of redisplacement requiring remanipulation after ED reduction compared with theater reduction (32.1% vs 11.1%, p = 0.048) [2]. In contrast, two other studies found no significant difference in redisplacement rates between treatment settings [3,4]. Differences in fracture selection, outcome definitions, and follow-up periods prevented quantitative pooling.

Repeat Intervention and Subsequent Treatment

All comparative studies reported repeat procedures or subsequent intervention, although definitions varied. Three studies found no significant difference between ED and theater pathways, whereas one study reported a higher rate of repeat manipulation after ED reduction [1-4]. In observational ED cohorts, subsequent GA in the operating theater was required in approximately 16% of children following attempted ED reduction [5]. Implementation of a structured ED pathway was associated with fewer failed ED manipulations, although causal interpretation was limited by the before-and-after design [6].

Cast Quality

Three studies evaluated cast quality using radiographic indices, including the cast index, padding index, canterbury index, or gap index. No significant differences were observed between ED and theater groups, and no consistent association was identified between cast quality measures and treatment failure [2-4].

Procedural and Anesthetic Complications

Complication reporting was limited. One study reported no cases of compartment syndrome in either treatment group. In contrast, another study reported overall complication rates of 16.9% after ED sedation and 13.3% after theater GA, with no significant difference (p = 0.185) [1,4]. Most reported events were minor, particularly nausea and vomiting. The available evidence was insufficient to determine comparative safety.

Admission and Length of Stay

ED management was associated with shorter hospital stays and lower admission rates in the comparative evidence. One study reported hospital admission in 12% of ED-managed children compared with 100% of theater-managed children (p < 0.0001), with a substantially shorter mean length of stay in the ED group (139 vs. 1,452 minutes, p < 0.0001) [1]. Similar trends were observed in observational data, although comparisons were limited by differences in treatment pathways [5].

Time to Treatment and Resource Utilization

Studies consistently demonstrated reduced resource use with ED management. ED reduction was associated with shorter time to manipulation, reduced theater utilization, and potential cost savings compared with theater-based management [1,5,6]. Economic estimates from observational studies were based on modeling assumptions and should be interpreted cautiously [5,6].

Predictors of Unsuccessful ED Reduction

The main predictors of ED treatment failure were assessed in one prognostic study. Subsequent theater-based GA was associated with delayed presentation (odds ratio (OR) 12.9, 95% CI 3.0-54.7), non-distal fracture location (OR 7.5, 95% CI 2.4-23.8), and increasing age (OR 1.13 per year, 95% CI 1.01-1.26) [5]. Another study reported that off-ended distal radius fractures frequently failed ED manipulation and required theater management [6].

Effects of ED Pathway Implementation

A single before-and-after study evaluated implementation of a BOAST-aligned ED pathway. Following pathway introduction, ED manipulation was attempted more frequently (31.9% vs 83.9%, p < 0.00001), and failure rates decreased (47.8% vs 13.7%, p = 0.0013) [6]. However, concurrent changes in clinical practice and the COVID-19 pandemic period limited attribution of these improvements solely to the pathway.

Overall, evidence suggests that ED procedural sedation can achieve acceptable outcomes for selected pediatric forearm fractures, with potential reductions in admission, treatment delay, and resource utilization. However, conclusions remain limited by observational study designs, selection bias, heterogeneous protocols, and inconsistent outcome reporting.

Limitations

This review has several limitations. All included studies were observational, mostly retrospective, and limited by small sample sizes and potential confounding by indication, as more complex fractures were often managed in the operating theater. Variation in sedation protocols, fracture characteristics, reduction criteria, imaging availability, operator experience, and outcome definitions limited comparability between studies. Adjusted analyses were uncommon, and functional outcomes, patient-reported outcomes, and detailed adverse-event data were frequently unavailable. At the review level, only six studies met the eligibility criteria, thereby preventing meta-analysis and formal assessment of publication bias. The lack of meta-analysis reflects substantial clinical and methodological heterogeneity rather than a limitation of the review methodology.

## Conclusions

ED procedural sedation is a potential approach to managing selected pediatric forearm fractures requiring closed reduction. Its use should be guided by appropriate patient selection, local expertise, available resources, and structured clinical pathways. Current uncertainty highlights the need for careful consideration of treatment setting, procedural safety, and follow-up requirements when choosing between ED and theater-based management. Further research with more consistent methodology and robust outcome assessment is required to better define the optimal approach.
